# Elevated CO₂: a double‐edged sword for plant defence against pathogens

**DOI:** 10.1111/nph.70048

**Published:** 2025-03-13

**Authors:** Rosa Sanchez‐Lucas, Estrella Luna

**Affiliations:** ^1^ Birmingham Institute of Forest Research, School of Biosciences University of Birmingham Birmingham B15 2TT UK

**Keywords:** atmospheric carbon dioxide, climate change, food security, forest resilience, plant immunity, plant pathogens

## Abstract

This article is a Commentary on Bredow *et al*. (2025), **246**: 2718–2737.

Human activities have caused atmospheric carbon dioxide (CO₂) concentrations to double since the onset of the industrial era, with levels continuing to rise at an unprecedented rate. This dramatic increase has far‐reaching consequences for plant physiology, ecosystems, and global agriculture. While rising CO₂ may enhance plant growth through the well‐known ‘CO₂ fertilisation’ effect (Ainsworth & Long, 2021), its influence on plant health is far more complex. Recent research has demonstrated that elevated CO₂ (eCO₂) can alter plant immune responses to pathogens, with highly variable outcomes. Reports indicate that eCO₂ can enhance, suppress or have no effect on plant resistance, depending on the specific host–pathogen interactions, environmental conditions, and pathogen lifestyles (Bazinet *et al*., 2022; Li & Ahammed, 2023; Sanchez‐Lucas *et al*., 2023; Smith & Luna, 2023). This variability underscores the need to better understand the interplay between eCO₂ and plant immunity to predict the potential impacts of climate change on plant health. Plant diseases, caused by a wide range of pathogens, are among the most significant threats to global food security, biodiversity, and forest health. The spread and virulence of plant pathogens are already being shaped by climate change, making it critical to investigate how rising CO₂ interacts with plant defence mechanisms. The study by Bredow *et al*. published in this issue of *New Phytologist* (2025; pp. 2718–2737) takes an important step towards addressing this knowledge gap. By evaluating how eCO₂ affects soybean in response to three distinct pathogen lifestyles (biotrophic, hemibiotrophic, and necrotrophic), the research provides a valuable framework for understanding the contrasting effects of eCO₂ reported in previous studies. Crucially, Bredow *et al*. explore the influence of eCO₂ under a concentration of 550 ppm, which reflects realistic projections for atmospheric CO₂ levels by 2050 (Hamdan *et al*., 2023). This approach enhances the relevance of their findings for understanding plant–pathogen dynamics in the near future, making their results directly applicable to ongoing discussions about the impacts of climate change on agriculture and ecosystems.

*Understanding the broader implications of eCO_2_ on plant performance remains a significant challenge due to the contrasting effects reported across diverse studies.*

## Novel findings addressing crucial gaps

In their study, Bredow *et al*. investigate how eCO₂ influences soybean physiology and disease outcomes, offering new insights into the complex interplay between plant growth, immune responses, and pathogen lifestyles. Consistent with previous research (Sanchez‐Lucas *et al*., 2023), the authors observed that eCO₂ boosted photosynthetic rates and biomass in soybean while decreasing stomatal conductance, density, and aperture (Soba *et al*., 2020). Nonetheless, gene expression analysis uncovered unexpected results: Certain photosynthesis‐related genes were downregulated despite the elevated photosynthetic performance, indicating a more complex regulatory network than the straightforward ‘fertilisation effect’ might suggest.

**Fig. 1:**
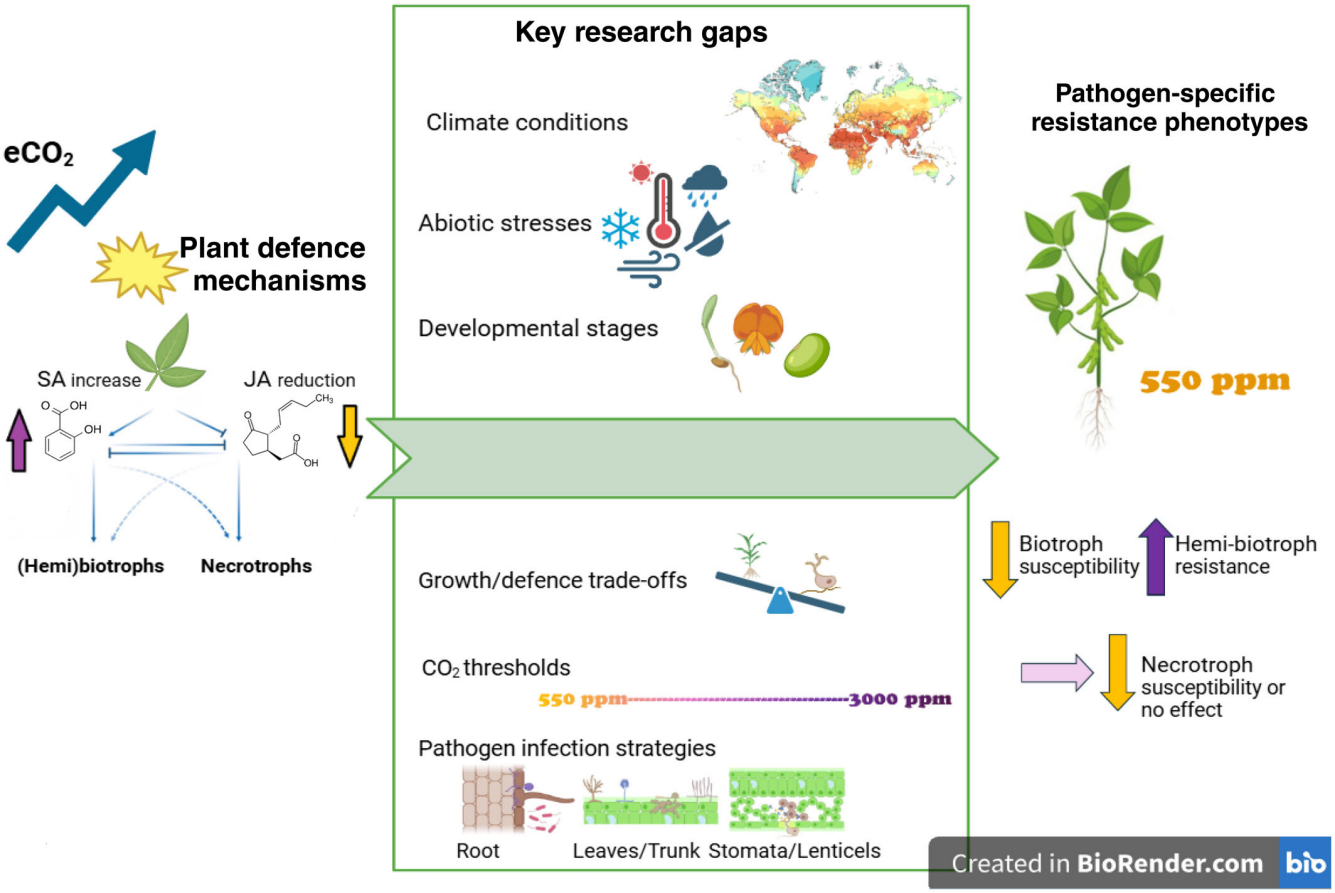
Schematic representation showing the effects of elevated CO₂ (eCO₂) on plant defence mechanisms and resistance phenotypes. Salicylic acid (SA) and jasmonic acid (JA)‐mediated defences regulate resistance to biotrophic, hemibiotrophic, and necrotrophic pathogens, with eCO₂ altering these responses. Key research gaps are highlighted. Pathogen‐specific resistance phenotypes reported in (Bredow *et al*., 2025; pp. 2718–2737, this issue of *New Phytologist*) vary under eCO₂ (550 ppm), with increased susceptibility to biotrophs, mixed responses to necrotrophs, and enhanced resistance to hemibiotrophs. Created in BioRender (https://BioRender.com/z04f308).

A key strength of the paper lies in its pathogen‐specific approach, covering bacterial, viral, fungal, and oomycete pathogens with different lifestyles. Under eCO₂, soybeans displayed enhanced resistance to the hemibiotrophic bacterium *Pseudomonas syringae* pv. *glycinea* (Psg). This was partly explained by reduced stomatal penetration and modest increases in salicylic acid (SA), although SA levels were less pronounced than in studies where strong SA‐dependent defences were reported (Williams *et al*., 2018). By contrast, soybeans became more susceptible to the biotrophic viral pathogens *Bean pod mottle virus* (BPMV) and *Soybean mosaic virus* (SMV). For soil‐borne pathogens, the study revealed increased susceptibility to the necrotrophic fungus *Fusarium virguliforme*, responsible for sudden death syndrome (SDS). Meanwhile, no major changes were observed in susceptibility to the necrotrophic oomycete *Pythium sylvaticum*, although infection by *Pythium* suppressed the growth advantages caused by eCO₂. Transcriptomic analyses unravelled that even without pathogen challenge, eCO₂ upregulated genes linked to basal immunity and jasmonic acid (JA) signaling. Surprisingly, this did not improve resistance against *F. virguliforme*, despite JA‐dependent defences normally conferring protection against necrotrophs. Therefore, this study highlights the pathogen‐specific effects of eCO₂ on soybean immunity, emphasising the complexity of plant–pathogen interactions under eCO_2_. The results of this paper highlight the context‐dependent nature of eCO₂‐induced immune responses and underscores the need for continued investigation into the mechanisms underlying these multifaceted interactions (Fig. 1).

## Broader implications and cross‐species perspectives

Understanding the broader implications of eCO₂ on plant performance remains a significant challenge due to the contrasting effects reported across diverse studies. While much is known about the trade‐offs between energy allocation to growth vs defence, less is understood about whether increased growth under eCO₂ might divert resources away from the effective activation of resistance responses. Considering that eCO₂ has been shown to enhance growth rates, it is crucial to study how these phenotypes influence the activation of defence mechanisms. Studies, such as Bredow *et al*., our own research (Sanchez‐Lucas *et al*., 2023), and others, have begun to address this gap focusing on the physiological impacts of eCO₂. On the one hand, eCO₂ is widely recognised for its positive effects on plant growth and biomass accumulation. On the contrary, it has been shown to disrupt carbon‐to‐nitrogen (C : N) ratios in leaves, potentially compromising plant nutritional quality (Jobe *et al*., 2020). Furthermore, our research on oak seedlings revealed that while eCO₂ promoted shoot biomass and photosynthetic rates, it simultaneously reduced root growth – a phenomenon also observed in some legumes (Soba *et al*., 2020). This reduction in root growth under eCO₂ could limit nutrient acquisition, exacerbating the C : N imbalance and potentially hindering the biosynthesis of key defensive compounds. These findings suggest that the ‘CO₂ fertilisation effect’ is far more complex than initially thought. Enhanced growth under eCO₂ may come at the cost of altered immune regulation, which could ultimately contribute to the observed variability in pathogen susceptibility. To address these challenges, further research is needed to unravel the mechanisms by which increased growth under eCO₂ influences plant defence pathways, and whether this trade‐off could leave plants more vulnerable to pathogen attack.

Existing literature categorises the effects of eCO₂ on plant–pathogen interactions as positive, negative, or neutral, underscoring the highly context‐dependent nature of these interactions. This variability makes it challenging to generalise plant responses across systems, pathogen lifestyles, and host species. For instance, Bredow *et al*. demonstrate how eCO₂ influences soybean defence responses, particularly through SA‐mediated pathways. Interestingly, despite an increase in SA levels typically associated with defence against biotrophs, soybean susceptibility to certain biotrophic pathogens, such as viruses, was heightened. Moreover, our research on oak seedlings revealed that eCO₂ increased susceptibility to the biotrophic fungal pathogen *Erysiphe alphitoides*, the causal agent of oak powdery mildew (Sanchez‐Lucas *et al*., 2023). These findings align with other studies that have reported enhanced SA‐dependent defences and increased SA accumulation under eCO₂. However, the enhanced susceptibility to biotrophic pathogens observed in both systems suggests that additional factors may override the expected SA‐mediated resistance. For instance, interactions with signaling pathways under the control of the plant hormone abscisic acid (ABA), highly controversial on its role in defence (Stevens *et al*., 2023) could alter the final resistance outcome. This is crucial because, while significant progress has been made in understanding eCO₂ effects on necrotrophic pathogens, much less is known about its influence on biotrophic pathogens. The findings by Bredow *et al*. contribute valuable insights to this underexplored area, but the emerging picture remains complex and contradictory. For example, soybean susceptibility to BPMV increased under eCO₂, yet in other systems, such as tomato, eCO₂ was associated with enhanced resistance to certain viruses (Huang *et al*., 2012). This variability highlights the pressing need for more targeted research into biotrophic pathosystems to disentangle the regulatory mechanisms involved and to identify the conditions under which eCO₂ enhances susceptibility or resistance. The context dependence of these interactions, as noted in our review paper focused on fungal pathogens, reinforces the idea that factors, such as pathogen lifestyle, host species, microbial community composition and environmental conditions, all contribute to shaping plant responses to eCO₂.

## Forward‐thinking perspective: a world under climate change

Climate change poses a multifaceted threat to global food security, biodiversity, and ecosystem stability, with rising atmospheric CO₂, heat waves, and drought among the most significant stressors. These interconnected factors profoundly influence plant health and productivity, making it essential to explore their combined effects on plant–pathogen dynamics. While studies incorporating additional abiotic stresses, such as heat and drought, are particularly important, they remain limited. While eCO₂ often enhances water use efficiency and mitigates some drought‐related stress, this combination has also been shown to increase susceptibility to pathogens. For example, one study in maize demonstrated that necrotrophic fungal infections worsened under combined eCO₂ and drought conditions (Vaughan *et al*., 2016). Similarly, although eCO₂ may alleviate certain heat‐induced physiological changes, this may not consistently translate to improved resistance against pathogens, as outcomes are highly species‐ and system‐dependent. These interactions highlight the need to investigate how multiple stresses interact to shape disease outcomes in both natural ecosystems and agricultural systems.

The study by Bredow *et al*. has resolved a significant part of this complex puzzle by evaluating the effects of eCO₂ on soybean interactions with pathogens of diverse lifestyles. Moreover, their integration of physiological, molecular, and transcriptomic analyses reveals the intricate interplay between plant defence pathways, growth responses, and environmental factors. These findings pave the way for future research aimed at unveiling the combined effects of eCO₂ and other abiotic stresses on plant immunity and resistance phenotypes. As we look towards a future shaped by escalating climate change, studies like this are critical for guiding the development of resilient crops and sustainable management strategies, ensuring plant health and productivity in increasingly hostile conditions.

## Disclaimer

The New Phytologist Foundation remains neutral with regard to jurisdictional claims in maps and in any institutional affiliations.
